# WER-Net: A New Lightweight Wide-Spectrum Encoding and Reconstruction Neural Network Applied to Computational Spectrum

**DOI:** 10.3390/s22166089

**Published:** 2022-08-15

**Authors:** Xinran Ding, Lin Yang, Mingyang Yi, Zhiteng Zhang, Zhen Liu, Huaiyuan Liu

**Affiliations:** 1 School of Information Science and Engineering, Shandong University, Qingdao 266237, China; 2 Institute of Frontier and Interdisciplinary Science, Shandong University, Qingdao 266237, China

**Keywords:** computational spectrometer, wide-spectrum encoding, convolutional neural network, hierarchical optimization

## Abstract

The computational spectrometer has significant potential for portable in situ applications. Encoding and reconstruction are the most critical technical procedures. In encoding, the random mass production and selection method lacks quantitative designs which leads to low encoding efficiency. In reconstruction, traditional spectrum reconstruction algorithms such as matching tracking and gradient descent demonstrate disadvantages like limited accuracy and efficiency. In this paper, we propose a new lightweight convolutional neural network called the wide-spectrum encoding and reconstruction neural network (WER-Net), which includes optical filters, quantitative spectral transmittance encoding, and fast spectral reconstruction of the encoded spectral information. The spectral transmittance curve obtained by WER-net can be fabricated through the inverse design network. The spectrometer developed based on WER-net experimentally demonstrates that it can achieve a 2-nm high resolution. In addition, the spectral transmittance encoding curve trained by WER-Net has also achieved good performance in other spectral reconstruction algorithms.

## 1. Introduction

Due to the good acquisition ability of target spectral information and image information, imaging spectrometers are widely used in remote sensing, medical, industrial, aerospace, and other fields. The first spectral detection was made by Isaac Newton, who used a triangular prism to divide sunlight into a rainbow-colored pattern. Traditional spectrometers are bulky, which has hindered them from achieving wider application in scenarios such as in satellites, drones, and handheld platforms. The computational spectroscopy technology based on the principle of compressed sensing (CS) was proposed [1], and the spectrometer, which used wide spectral coding, became a hot topic in research because it could increase the signal-to-noise ratio (SNR). Many computational methods have been introduced into spectral detection. It has become possible to realize portable sensing systems [2,3].

Tao et al. proposed restricted isometry property (RIP) to guide the construction of the sensing matrix (i.e., the product of the measurement matrix of optical filters transmittance and the sparse basis matrix) [1]. Based on RIP, scholars have successively improved the original algorithm and proposed OMP [4], StOMP [5], and TMP [6]. Donoho proposed the non-correlation criterion of the measurement matrix constructed by the filter transmittance curve [7], which supplies researchers with an easy way to construct the sensing matrix. The performance of the computational spectrometer is strongly related to the non-correlation of its encoding filters. However, there is a full-beam relationship between the transmittance curve and the filter structure design parameters. The mass-production and then try to select only a few of them that can meet the in non-correlation criterion is unacceptable. The low encoding efficiency also explains why the reconstruction accuracy of the spectrometer obtained by this method is largely limited.

Recently, deep neural networks are being applied to the field of spectral reconstruction [8,9,10,11], but a lightweight convolution neural network (CNN) has few been reported in this arena. Kulkarni pioneered work in the field of image reconstruction by using CNNs [9]. Hao [8] applied neural networks in computational spectroscopy, and it is worth noting that the architecture is featured with all fully connected (FC) layers. The work of these authors inspired us to propose a CNN with both filter-encoding and spectral reconstruction capabilities. A CNN is featured with a few parameters that can increase efficiency. In addition, its virtue of weighting parameter sharing and the use of sparse connections of local receptive fields enables the CNN to have better performance in the training consumption and recovery accuracy of computational spectral reconstruction. WER-Net uses an FC layer without offset to encode the incident spectrum and reconstruct the encoded information afterward by combining convolutional layers and FC layers. The training data for this network is obtained from CAVE [10] and ICVL [11], and the transmittance curve of a group of filters and its corresponding reconstruction network can be completed after training.

The rest of this paper is organized as follows. In Section 2, we describe WER-Net’s architecture and methodology. Section 3 describes WER-Net’s training procedure. Experiment results are reported in Section 4. Finally, Section 5 concludes the paper.

## 2. Methodology

Neurons are used as the basis of deep neural networks to simulate the working process of biological neural networks, and the most commonly used neuron model is the M-P neuron model proposed by McCulloch [12]. Hopfield used neural networks to solve NP-hard problems for the first time [13], which helped drive the rapid development of neural networks. After this, LeCun proposed LeNet-5, the standard CNN [14], which greatly contributed to the development of the CNN. Since then, deep neural networks have flourished in a variety of fields. Among them, Kulkarni applied it to the field of image compression and reconstruction [9], opening up the use of deep neural networks to solve CS problems. Zhang then applied feed-forward neural networks to the field of spectral reconstruction [8].

WER-Net is composed of the encoding network and the reconstruction network. On the one hand, for the encoding network, because the spectral sampling process of the wide spectral encoding filter and the input operation process of the fully connected layer without bias are defined by matrix multiplication, a layer of fully connected layers without bias can be used to simulate the wide spectral coding filter and obtain the corresponding spectral transmittance curve. On the other hand, the reconstruction network simulates the process of solving the CS problem. According to the universal approximation theorem, the NP-hard problem of solving the CS problem can be solved by using more than three layers of neural networks. After adjusting the network structure to achieve the balance between solving accuracy and efficiency, we decided to use two layers of fully connected layers and three layers of a convolutional layer to realize the solving process.

**Figure 1:**
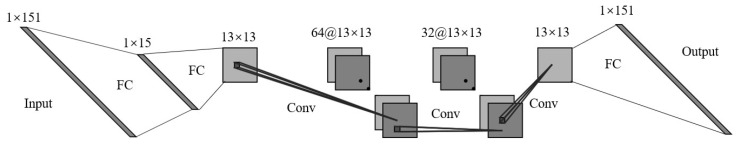
The network architecture of WER-Net.

The network architecture of WER-Net proposed in this paper is stated as follows. The first layer uses sampling of a fully connected layer without offset to simulate optical encoding filters, followed by spectral reconstruction by using two fully connected layers and three convolutional layers. Among them, except for the first and last layers, the ReLU function is used as the activation function. The above structure can also be described as: (FC without offset)-FC-Rule-(Conv-Rule)3-FC. To the best of our knowledge, this is the first-time convolutional layers are used in spectral reconstruction. The network structure diagram is shown in Figure 1.

### 2.1. Engineered Loss-Function

The input and output of WER-Net are 400~700 nm@2nm spectral information matrix, and the training goal is

(1) $$\Theta =argmin{\left|\left|S-\hat{S}\right|\right|}_{2},$$

where *Θ* represents the set of parameters in each layer of the neural network, *S* is the spectral information matrix of the input, and $\hat{S}$ is the spectral reconstruction matrix of the output.

Equation (1) simply indicate the nature of the computational spectrometer. However, it has no regulating ability on the optical filters. After rounds of training, it ends up with an ideal spectral transmittance curve that fully complies with the non-correlation criterion. That brings us to a severe problem for the fabrication of optical filters because transmittance curves in the real world lack the spectral diversity of the ideal ones. That means researchers can never have the result they want so they adopt the random Gaussian matrix as the measurement matrix [15,16]. This may make for impressive performance with regard to reconstruction accuracy, but the overly random filters cannot be produced.

To address this problem, with the development of deep learning technology, many reverse design methods dedicated to achieving spectral responses have been presented [17,18,19]. However, due to poor generalization, it is still difficult to achieve accurate reverse design for some extreme requirements. The good news is that as long as the spectral transmittance curve is smooth enough, the above methods can give good reverse design results. Therefore, in order to obtain filters that are characterized by technological feasibility while taking the requirements of Equation (1) into consideration, we make the weight parameters of the first FC layer as smooth as possible on each line. Consequently, we use the following loss function:

(2) $$loss={\left|\left|S-\hat{S}\right|\right|}_{2}+\delta \underset{i}{\sum }\left|\omega_{i}-\omega_{i+1}\right|.$$

To circumvent the slow speed of the circulation operation, we take the values of the first to *n*th columns of the weight matrix *W* to form the matrix *W′*, and take out the former n-1 columns of the *W* to form the matrix *W″*. The equivalent form of Equation (2) is shown in Equation (3):

(3) $$loss={\left|\left|S-\hat{S}\right|\right|}_{2}+\delta \sum \left|W^{\prime }-W^{\prime \prime }\right|.$$

The optical filter transmittance curve corresponding to the first FC layer without offsets trained in this way has good engineering characteristics and can be fabricated.

### 2.2. FC Layer in Encoding and Hierarchic Optimization

**Figure 2:**
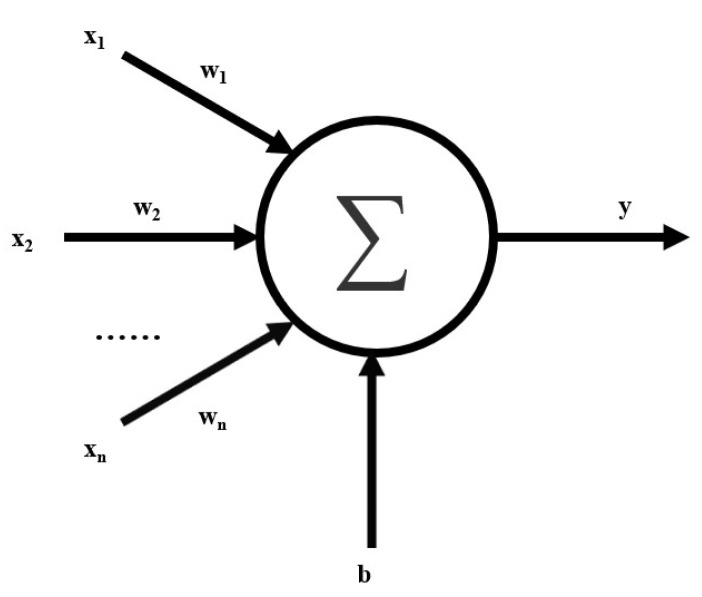
M-P neuron model.

The structure of the M-P neuron model is shown in Figure 2.

The neuronal model in Figure 2 can be represented as the following equation:

(4) $$y=f\left(\overset{n}{\underset{i=0}{\sum }}\omega_{i}x_{i}+b\right)=f\left(W^{T}\cdot X+b\right),$$

where f(·) represents the activation function, $W=\left[w_{0},w_{1},\ldots ,w_{n}\right]$ for the weight value, and $X=\left[x_{0},x_{1},\ldots ,x_{n}\right]\;$ for the input. For an FC layer composed of multiple neurons, it can be expressed as Equation (5) when the role of the activation function is not considered:

(5) $$Y=W^{T}\cdot X+B.$$

For wide-spectrum coding filters, the coding of spectra is as follows:

(6) $$E=\int_{\lambda_{1}}^{\lambda_{2}}T\left(\lambda \right)S\left(\lambda \right)d\lambda ,$$

where *E* represents the output of the filter encoded spectral signal, *T*(*λ*) represents the spectral transmittance of the filter, and *S*(*λ*) represents the incident spectral signal.

Discretize Equation (6), and we have

(7) $$E=\overset{n}{\underset{i=0}{\sum }}T_{i}\left(\lambda \right)S_{i}\left(\lambda \right).$$

Discretize the incident spectral signal and the spectral transmittance, and the encoded spectral information can be described in matrix form:

(8) $$E=T^{T}\cdot S.$$

If the incident spectral signal matrix *S* in Equation (8) is taken as the input *X* represented in Equation (5), and the offset term *B* is zeroed, then the output *Y* of Equation (5) equals *E* of Equation (8). In a nutshell, an FC layer without offset can simulate the process when incidental light passes through the optical filter, hence being encoded. In the proposed WER-Net, this method is used to design the wide-spectrum encoding filter.

**Figure 3:**
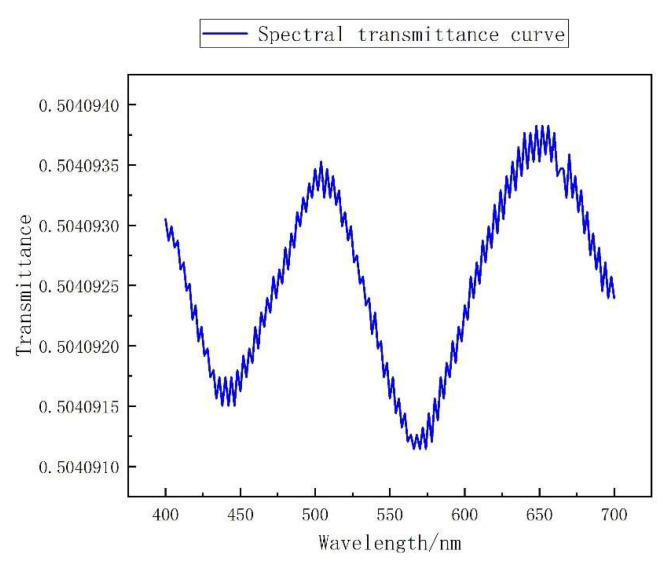
Jittering spectral transmittance curve.

According to the principle of CS, the greater the non-correlation of the sampling matrix, the more object information can be obtained. Therefore, based on the aforementioned loss function, it can be judged that the training logic of the first FC layer without offset is to take into account the non-correlation and production process limitations. However, in the scenario following a certain number of training rounds, the spectral transmittance curve begins to jitter on the whole spectrum range, as shown in Figure 3. It improves with non-correlation, but it brings great difficulties to the fabrication process of the filter.

Therefore, this paper proposes a hierarchical optimization method for WER-Net. Specifically, under the condition that the filter transmittance curve shows sufficient diversity, we stop the training of the first FC layer parameters, and retain it, and on this basis, the training of the full connection layer and convolutional layer parameters of the reconstruction part is continued. The hierarchical optimization method helps to maintain the technical feasibility of filter fabrication and also reduce the time and spatial complexity of training.

### 2.3. Spectral Reconstruction Using CNN

Deep neural networks have extremely powerful expression capabilities. They require only a single hidden layer and a small number of neural units to fit functions of any complexity with high precision [20,21,22,23]. To the best of our knowledge, some deep neural networks have been introduced into computational spectrometry [8,15,16]. However, the application of CNNs in computational spectrometry is rarely reported. Moreover, this elucidates the major difference between WER-Net and other deep neural networks in spectrum reconstruction.

The structure of the convolutional layer can be expressed in Equation (9),

(9) $$y=\overset{n}{\underset{i,j}{\sum }}\theta_{i,j}x_{i,j}+b,$$

where *θ*_(*i,j*) represents the convolutional kernel element size of column *j* in row *i, x*_(*i,j*) represents the element size of column *j* in row *i*, and *b* is the deviation.

Although the common feedback-forward neural network in [8] can be used to calculate the spectral reconstruction, severe challenges with regard to time and accuracy performance remain.

On the one hand, the feedback-forward neural network has a great number of parameters to be trained and stored. As a result, it needs a gigantic training dataset and becomes incompatible with embedded systems. If there are 100 hidden neurons in the first FC layer when applying neural networks for computational spectral reconstruction, thousands of weighting parameters would need to be updated in the first FC layer alone. This would lead to a drastic increase in both system storage demands and the training dataset volume. Otherwise, we have an inefficient decoding network which cannot be applied to scenarios like in situ measurement or real-time measurement. In addition, this bulky network cannot be easily embedded on many types of hardware.

On the other hand, the feedback-forward neural network lacks the ability to share its weighting parameters which would result in low efficiency in data fitting. Taking a random curve for instance, there could be a lot of diversities in this curve. It could be a high-order function, a random noise, an aperiodic impulse curve, etc. In order to match this curve, the feedback-forward neural network would perform a crude and time-consuming fitting process. However, in the real world, most of the spectral curves are demonstrating gradual changes. It means the neighboring wavelengths have a relatively good correlation property. Obtaining this property would benefit the neural network greatly. CNNs solve the above problems by virtue of their weighting parameter sharing and the use of sparse connections of local receptive fields [14], which makes CNNs have better performance in the training consumption and recovery accuracy of computational spectral reconstruction.

### 2.4. Dataset Augmentation

To train the WER-Net, we used a total of 1,650,000 spectral data from the CAVE and ICVL. Both are 10-nm resolution data in the range of 400 to 700 nm.

In order to achieve the application of the network to a high-resolution spectrometer, this article conceives the idea to use some interpolation method to augment the 10-nm resolution database to a higher resolution one. For instance, to achieve 2-nm resolution, the least squares fitting is applied to augment the data of CAVE and ICVL, and the data of 31 × 1,650,000 is processed into 151 × 1,650,000 data. In the later experiment demonstration, the idea is verified.

### 2.5. Activation Function

At present, the mathematical principles of computational spectral algorithms, whether they are traditional GPSR [24], or OMP [4], or computational spectral algorithms based on deep learning, can be explained by CS theory.

Considering the signal *x*∈R^N, the measurement process can be expressed as follows:

(10) y=Φx,

where the matrix *Φ*∈R^(M × N) is called the measurement matrix, and *y*∈R^M is the measurement vector. The encoding process is completed by the measurement process. The process of recovering the original signal from the measured value by means of a computational reconstruction is termed “reconstruction”. When *Φ*∈R^(M × N), Equation (10) is an underdetermined entity. The linear inverse problem is a pathological problem. Solving a problem means recovering more information with less information, which is clearly inconsistent with Nyquist’s sampling theorem. Based on the CS theorem [25,26,27], when both the signal and the measurement matrix meet certain conditions, even if M << N the original information can be recovered.

The process of solving the compressive perception is an NP-hard problem, instead of a simple linear problem. Referring to the universal approximation theorem, an activation function is needed to enable a neural network to fit a nonlinear problem. The nonlinearity of activation function needs delicate consideration.

**Figure 4:**
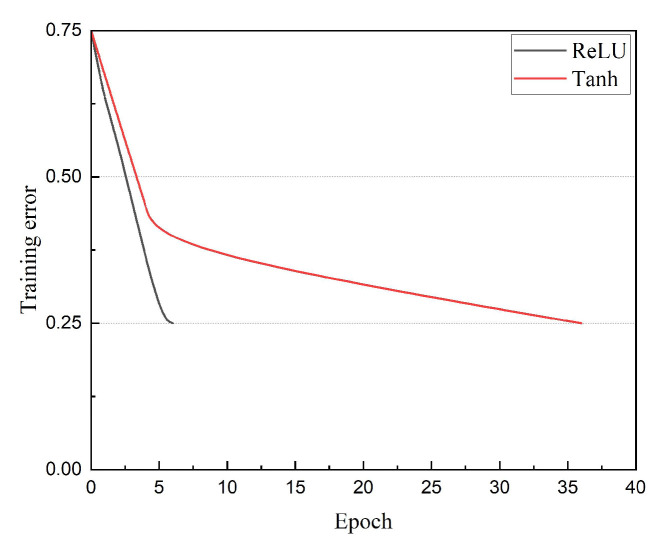
Comparison of network training speed using ReLU and Tanh.

It is widely acknowledged that the nonlinear performance of the ReLU function is superior to that of functions such as Tanh, as shown in Figure 4, and the training error rate of a four-layer convolutional neural network by using the ReLU function reaches 25% faster than that of an equivalent network with a Tanh activation function [28]. Therefore, the activation function used by WER-Net is the ReLU function.

## 3. WER-Net Training

### 3.1. Level-1 of Hierarchical Optimization

**Figure 5:**
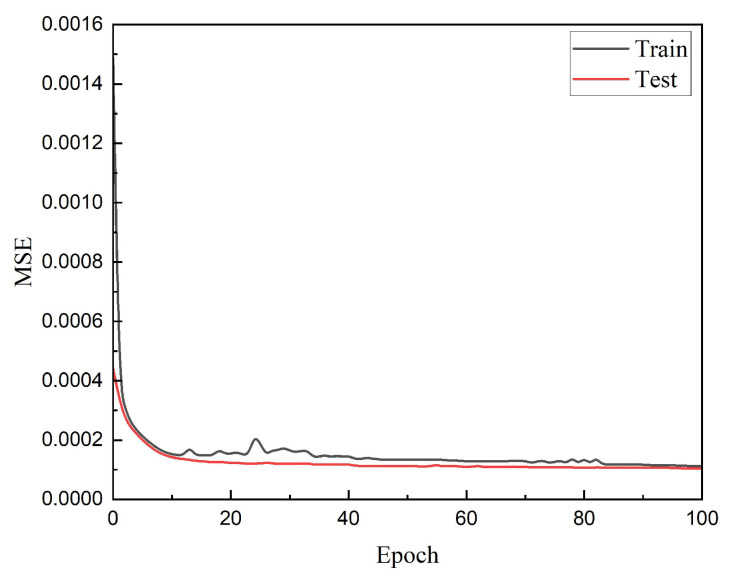
Training error and test error of level-1 of hierarchical optimization.

The training model used 151 × 1,650,000 data obtained by least squares interpolation. The data is divided into a training set and a test set by 10:1 ratio. Because the dataset is so large, if each piece of data is trained one by one, the cost of time and hashing power will be unbearable. Therefore, to ensure that the reconstruction accuracy is not lost, we have adopted the batch training method. Figure 5 shows the training error and test error for each training session. By the time the training reaches 10 times, the network has achieved preliminary convergence, the recovery accuracy of the training set has reached 1.5 × 10^−4^, and the recovery accuracy of the test set has reached 1.4 × 10^−4^.

### 3.2. Level-2 of Hierarchical Optimization

**Figure 6:**
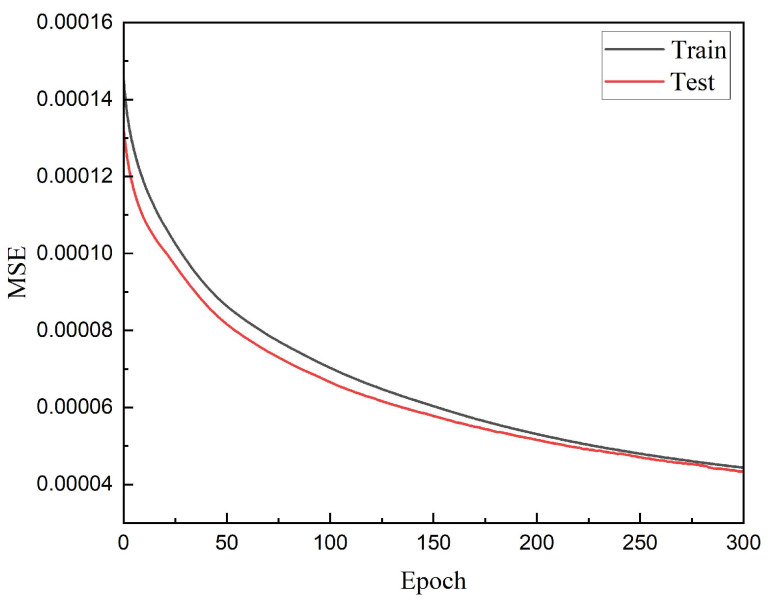
Training error and test error of level-2 of hierarchical optimization.

At this time, the smoothing characteristics of the obtained spectral transmittance curve are also very good, so the model is used as a pre-trained model for hierarchical optimization. Figure 6 shows the training error and test error of training, when training is getting close to 300 times. The accuracy improvement is very slow, although there is no overfitting, and the wasted computing power resources of continued training and the benefits of accuracy improvement are not rewarding. At this time, the training is stopped, and the recovery accuracy of the training set reaches 4.4 × 10^−5^, and the recovery accuracy of the test set reaches 4.3 × 10^−5^. The training of WER-Net far exceeds the network in [8] in terms of convergence speed and recovery accuracy. Through the later experimental verification, the trained WER-Net achieves a 2-nm resolution.

**Figure 7:**
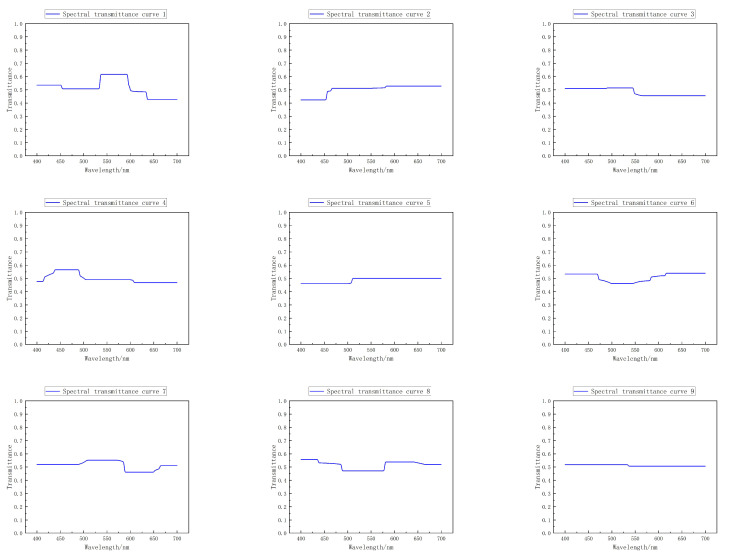

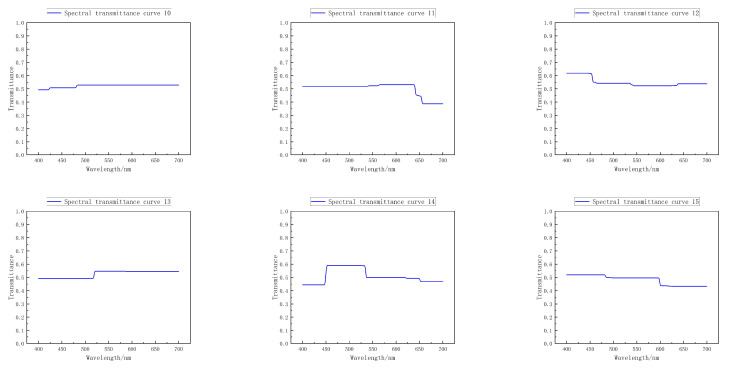
Spectral transmittance curves of all trained optical filters.

After the training, the spectral transmittance curve of the filter is obtained by invoking the FC layer parameter of the first FC layer without offset in the optimal network. As can be seen from Figure 7, the spectral transmittance curve of the filter is smooth, and it can be preliminarily judged that it can be reverse-designed and produced.

## 4. Experiment

### 4.1. Experimental Design and Results

**Figure 8:**
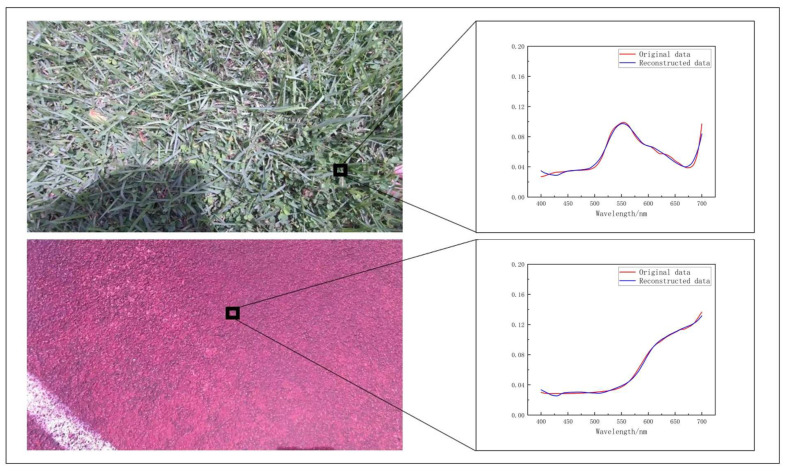
Reconstructed spectrum by WER-Net.

To simulate the manufacturing errors generated in the filter production process, a Gaussian random noise with standard deviations *σ* of 0.001 and 0.01 was introduced to the weighting parameters of the first FC layer. According to the Monte Carlo method, we used the spectral information of nearly a thousand real objects with 2 nm resolution captured by IspecField-HH spectrometer, and entered them into the WER-Net for encoding and reconstruction. Some of the results are shown in Figure 8. As a result, the mean squared error (MSE) of the reconstructed spectra can reach 9.374 × 10^−5^. The result is much similar to the optimal network test error, so it can be concluded that the WER-Net has a good performance on resolution of 2 nm spectrum reconstruction. Table 1 shows the critical performance values for spectral data reconstruction, including MSE, full width at half maximum (FWHM), peak amplitude error, peak wavelength position deviation, and reconstruction speed. The recovery speed is experimental data running on the Nvidia GeForce RTX2060 platform.

Finally, the spectral transmittance curve trained by WER-Net is fed into the inverse design network (IDN) to prove its practical application [29]. The MSE of the spectral transmittance curve obtained by IDN is 4.62 × 10^−2^, which proves that the spectral transmittance curve trained by WER-Net can be designed and fabricated. At the same time, WER-Net has strong robustness. Table 1 shows that WER-Net performs extremely well even with a certain level of noise. Therefore, WER-Net can accept the error within a certain range in the reverse design and production of the filter, which can be really applied to engineering.

### 4.2. Comparison with Other Algorithms

We selected two traditional CS algorithms and a deep learning algorithm, namely gradient projection sparse reconstruction (GPSR), orthogonal matching trace (OMP) and PCSED in [8]. The results of the comparison are shown in Table 2. The reconstruction operation using GPSR and OMP has been performed on the AMD Ryzen 5-3500U platform, and PCSED has been performed on the Nvidia GeForce RTX2060 platform.

For GPSR and OMP algorithms, the ideal random Gaussian matrix and the optical filter matrix of WER-Net are both adopted. As expected, the ideal non-correlated Gaussian matrix plays better both on MSE and speed than the ones with optical filter of WER-Net. However, the fabrication of random Gaussian matrix type filters can hardly be possible.

In contrast, the filter obtained by WER-Net not only can be used by WER-Net, but can also have a good performance with other spectral reconstruction algorithms like GPSR and OMP. Table 2 shows the spectral reconstruction performance. The reconstruction accuracy (MSE) of the GPSR algorithm can reach 2.20 × 10^−2^, and the reconstruction accuracy of the OMP algorithm can reach 4.63 × 10^−3^. Most impressively, the WER-Net has better performance both on MSE and reconstruction speed. The reconstruction accuracy of WER-Net is 208 times higher than GPSR, 38 times higher than OMP, and the reconstruction speed is only 0.48% of GPSR and 2.65% of OMP.

Compared with PCSED described in [26], which also has the ability of encoding and reconstruction, the reconstruction accuracy of WER-NET is 17.3 times higher than that of PCSED, and the reconstruction speed is only 35.7% of that of PCSED. This indicates that the convolutional layers of WER-Net play a difference.

## 5. Discussion

This paper presents a novel encoding and reconstruction artificial neural network called WER-Net, which is applied in a computational spectrum field. First, an FC layer without offset was constructed to simulate the encoding process of the spectrum, and then two FC layers and three convolution layers were architected to reconstruct the encoded spectrum. The open-source CAVE and ICVL database were then used for least square interpolation to obtain a virtual higher resolution training dataset. The database is used to train WER-Net to obtain the spectral transmittance and decoding network. In the encoding part, to acquire spectral transmittance, the regular terms and hierarchical optimization methods during training are used to make it more practical in engineering. In the reconstruction part, CNNs are used to reduce the network parameters and improve computational efficiency. Finally, experimental demonstration proves that the wide-spectrum encoding filter trained by WER-Net has universal applicability. The high-precision spectral reconstruction is successfully realized in traditional GPSR, OMP, and other algorithms. Moreover, the reconstruction accuracy of WER-Net is 208 times higher than GPSR, 38 times higher than OMP, and the reconstruction speed is only 0.48% of GPSR and 2.65% of OMP. WER-Net not only solves the drawbacks of high cost caused by the “mass production selection” tendencies of wide-spectrum encoding filters, but also greatly improves the reconstruction efficiency.

## Figures and Tables

**Table 1:** Mean value of WER-Net performance index.

	*σ* = 0	*σ* = 0.001	*σ* = 0.01
MSE	9.374 × 10^−5^	1.129 × 10^−4^	3.510 × 10^−4^
FWHM	0.986 nm	0.970 nm	1.136 nm
Peak amplitude error	1.452 × 10^−3^	1.255 × 10^−3^	2.821 × 10^−3^
Peak wavelength position deviation	0.25 nm	0.25 nm	0.5 nm
Reconstruction speed	343.89 μs	447.04 μs	378.22 μs

**Table 2:** WER-Net compares with other algorithms.

	GPSR (with Gaussian Matrix)	GPSR (with Filter Matrix of WER-Net)	OMP (with Gaussian Matrix)	OMP (with Filter Matrix of WER-Net)	PCSED	WER-Net
MSE	1.95 × 10^−2^	2.20 × 10^−2^	3.54 × 10^−3^	4.63 × 10^−3^	5.413 × 10^−4^	9.374 × 10^−5^
Reconstruction speed	21 ms	91.1 ms	7.4 ms	13.4 ms	963.37 μs	343.89 μs

## Data Availability

Not applicable.
